# Using Virtual Reality to Improve Apathy in Residential Aged Care: Mixed Methods Study

**DOI:** 10.2196/17632

**Published:** 2020-06-26

**Authors:** Dimitrios Saredakis, Hannah AD Keage, Megan Corlis, Tobias Loetscher

**Affiliations:** 1 University of South Australia Adelaide Australia; 2 Helping Hand Aged Care Adelaide Australia

**Keywords:** reminiscence, head-mounted display, apathy, cognitive aging, dementia, residential facilities, virtual reality

## Abstract

**Background:**

Apathy is a common symptom in neurological disorders, including dementia, and is associated with a faster rate of cognitive decline, reduced quality of life, and high caregiver burden. There is a lack of effective pharmacological treatments for apathy, and nonpharmacological interventions are a preferred first-line approach to treatment. Virtual reality (VR) using head-mounted displays (HMDs) is being successfully used in exposure- and distraction-based therapies; however, there is limited research on using HMDs for symptoms of neurological disorders.

**Objective:**

This feasibility study aimed to assess whether VR using HMDs could be used to deliver tailored reminiscence therapy and examine the willingness to participate, response rates to measures, time taken to create tailored content, and technical problems. In addition, this study aimed to explore the immediate effects between verbal fluency and apathy after exposure to VR.

**Methods:**

A mixed methods study was conducted in a sample of older adults residing in aged care, and 17 participants were recruited. Apathy was measured using the Apathy Evaluation Scale (AES), and verbal fluency was used as a proxy measure of improvements in apathy and debriefing interviews to assess feedback from participants. Side effects that can occur from using HMDs were also measured.

**Results:**

We recruited participants from a high socioeconomic status setting with a high education level, and the participation rate was 85% (17/20); most responses to measures were positive. Access to a wide range of freely available content and the absence of technical difficulties made the delivery of a VR reminiscence intervention highly feasible. Participants had improved semantic scores (*t*_14_=−3.27; *P*=.006) but not phonemic fluency scores (*t*_14_=0.55; *P*=.59) immediately after the intervention. Those with higher levels of apathy demonstrated the greatest cognitive improvements after a VR reminiscence experience, which was indicated by a strong positive relationship between the AES and semantic verbal fluency change scores postminus pre-VR (*r*=0.719; 95% CI 0.327 to 0.900; *P*=.003). All participants enjoyed the experience despite 35% (6/17) of participants experiencing temporary side effects.

**Conclusions:**

This study provides initial evidence that it is feasible to use VR with HMDs for therapy to treat symptoms of apathy in older adults in residential aged care. However, there is a need to closely monitor the side effects of HMD use in older adults. Further research is needed using an active control group to compare the use of VR with traditional forms of reminiscence therapy.

## Introduction

### Background

Apathy is one of the most common symptoms of neurological and psychiatric disorders such as dementia [1-3]. A lack of motivation is the defining feature of apathy, resulting in a reduction of goal-directed behavior [4]. Apathy contributes to a poorer quality of life and a faster rate of cognitive decline [5,6]. In addition, the risk of mortality in those with apathy increases 3-fold compared with those without apathy [7]. Those caring for people with apathy are also affected by reported higher caregiver burden compared with other neuropsychiatric symptoms [8,9].

A high prevalence of apathy of up to 84% has been reported in older adults in residential aged care with cognitive decline [10]. Healthy older adults can also experience symptoms of apathy in varying degrees [11,12]. Significant changes in life circumstances, for example, surviving natural disasters or being institutionalized, may reduce motivation, and increase symptoms of apathy [13]. Changes in specific brain regions have been associated with apathy, including frontal-subcortical structures [14,15]. However, the mechanisms of apathy are not fully understood.

Although symptoms of apathy may overlap with those of depression [16], apathy can also present as a separate clinical symptom [17], and there is increasing evidence supporting this distinction [18,19]. Symptoms of apathy can also differ depending on the type of dementia, with more severe symptoms found in the behavioral variant of frontotemporal dementia [20]. With little evidence supporting pharmacological treatment for apathy [21] and the side effects of current medications [22], alternative interventions are required.

Nonpharmacological approaches have demonstrated evidence in improving the levels of apathy, including music, art, cognitive stimulation, and reminiscence therapy [23]. Reminiscence therapy is commonly used in residential aged care and involves the discussion of a person’s past experiences involving the use of items including photographs, magazines, or music [24]. It can help maintain a person’s sense of identity and improve feelings of self-worth [25,26], an important aspect of improving apathy.

A recent Cochrane review found reminiscence therapy to be effective in some cases [27]. However, the effect sizes found were small and inconsistent. Little or no differences in cognitive outcomes were commonly found when comparing reminiscence therapy–treated groups with nontreatment groups (eg, standardized mean difference 0.11, 95% CI 0.00 to 0.23) [27]. Improving the delivery of tailored reminiscence therapy by increasing how realistic it is may increase the effectiveness of the therapy and the likelihood of attending sessions [28].

The use of technology in reminiscence therapy can provide access to a wide range of content via the internet and make it easy to tailor an intervention specifically for a person [29,30]. In a within-group study comparing the creation of a digital life storybook with a conventional life storybook for people with dementia, additional improvements were seen in the quality of life and autobiographical memory performance when the digital life storybook was used [30]. The traditional version in this study consisted of tangible prompts, including photos and other memorabilia, and the digital version consisted of a movie created and burned onto a DVD for playing on television. iPads have also been used in the delivery of individualized reminiscence therapy with positive results, including improvements in well-being [31,32]. Significantly higher levels of initiating speech and engagement were found using a touchscreen monitor compared with traditional reminiscence therapy using artifacts, including newspapers, books, and photos [33]. Virtual reality (VR) in the form of large-screen displays has also been used and provides an immersive experience [34]. The level of immersion can vary among technologies. iPads or computer screens are classified as nonimmersive, whereas large-screen displays are considered semi-immersive [35]. An increase in the level of immersion can increase the level of presence (the degree to which a person feels they are in the virtual environment) [36]. During a navigation task, an increased sense of presence using highly immersive 3D screens while wearing stereo glasses has been found to recruit additional brain areas compared with less immersive 2D screens [37].

VR using head-mounted displays (HMDs) provides a fully immersive, highly engaging, and realistic experience with the user closed off from external distractions. The use of HMDs is not limited to gaming and has infiltrated many areas, including medical training, rehabilitation, and therapy [38-41]. Advances in technology, production, and reduced costs have made HMDs widely accessible, with little training required to implement and use them. In patients who had previously not reported clinical benefits from other forms of psychotherapy, the use of VR using HMDs has been successful in treating post-traumatic stress disorder (PTSD), with a significant decrease in PTSD symptoms being found while comparing pretreatment symptoms with posttreatment symptoms (*d*=1.17) [42]. Other successful interventions using HMDs include reducing anxiety [43] and pain management of burn victims during wound care [44,45].

Recent research has found that older adults, including those with cognitive decline, can tolerate using HMDs and has reported positive experiences [46-49]. There has also been success in using HMDs for memory training in older adults with significant improvements in memory tests (*d*=.7) compared with a control group receiving music therapy [50]. Other uses of HMDs, including in older adults, have shown an increase in the performance of daily living activities in stroke victims [51] and success with vestibular rehabilitation in older adults with mild cognitive impairment [52]. These findings of HMD use in older adults suggest that expansion into other areas is possible. Research into the use of VR using HMDs in therapy for symptoms of neurological disorders is limited.

In addition to the lack of motivation in apathy [53], executive dysfunction has also been found to be associated with apathy [54,55]. Impairment in executive function is a key cognitive mechanism of apathy. Apathy is negatively associated with the performance of verbal fluency tasks [56]. Similarly, those with dementia and apathy have shown reduced performance on verbal fluency tasks, compared with those without apathy [57]. Specifically, associations have been reported between apathy and semantic fluency but not phonemic fluency [56,58]. Performance in verbal fluency tasks indicates executive control [59] and initiation [60]. These executive functions of control and initiation are reduced in those with apathy [61,62]. Apathetic participants demonstrated a preference for a VR task over a pen and paper task, as it provided a more engaging experience [63]. In addition, VR (using a projector screen with stereo glasses) has demonstrated the ability to stimulate autobiographical memories [34]. Therefore, VR may improve the performance of executive functions, both directly and indirectly.

Although there is evidence supporting the acceptability of using HMDs in older adults as previously mentioned, some people do experience side effects from using HMDs, including the occurrence of motion sickness such as that induced by air, sea, or land travel. Research on sickness symptoms while using HMDs in older adults has reported conflicting outcomes [64-66]. Many companies are starting to offer VR services for older adults in residential aged care [67-69]. However, the occurrence and prevalence of side effects of using VR HMDs with this age group are uncertain [70]. With the increased use of VR using HMDs in many areas, including older adults, awareness of any side effects from using this technology has become essential.

### Objectives

This study aimed to evaluate the feasibility of using tailored content created for viewing in a VR HMD to deliver reminiscence therapy to older adults in residential aged care. Along with establishing the feasibility of this study, the following parameters were also examined: availability and willingness to participate, response rates to measures used, the time needed to create tailored content, and technical problems. Verbal fluency was used as a measure of immediate effects on executive function after exposure to tailored content in VR, and associations with verbal fluency and apathy were also examined. A debriefing interview was used to assess enjoyment and obtain feedback from participants. Side effects of using HMDs were also examined using the Simulator Sickness Questionnaire (SSQ) combined with feedback from the debriefing interview.

## Methods

### Participants

Participants were recruited from a residential aged care facility in Adelaide, Australia. Potentially suitable participants were identified by senior staff at the residential aged care facility following the inclusion and exclusion criteria. A list of identified participants was then given to the researcher, and the researcher approached participants to request their participation in the study. The current health status of each participant was obtained from the nursing staff on duty before the participants were contacted. Out of 20 participants who were requested to participate in this study, 3 declined; 10 women and 7 men, between the ages of 72 and 95 years with a mean age of 87.3 years (SD 6.3), agreed to participate. All the participants were Australian, of whom 11 were born in Australia, 2 in the United Kingdom, 2 in Africa, and 2 in Europe. The participants born in the United Kingdom, Africa, and Europe had spent most of their lives in Australia. The participants came from a high socioeconomic background and most were well educated; the mean years of education was 13 (SD 3). According to the Psychogeriatric Assessment Scale (PAS), 10 participants had no or minimal cognitive impairment, 3 presented with mild impairments, and 4 had moderate impairments [71]. The mean score for the Apathy Evaluation Scale (AES) was 31.5 (SD 6.3). Using a cutoff score of 36.5 [72], 3 participants met the criteria for a diagnosis of apathy with the highest score being 46; 5 participants scored between 34 and 36. Medication history was available for 14 participants. The most common medication used was for depressive symptoms in 6 out of 14 participants (43%). Participants were excluded if their score on the PAS was 16 or higher indicating severe impairment; had significant neurological disorders; had other conditions, including agitation and aggression at a level that would make assessment difficult; or if their vision could not be corrected by glasses or glass frames that could not fit into the HMD. Informed consent was obtained from all the participants, and no honorarium was offered for participation. Ethics approval was obtained from the University of South Australian Human Research Ethics Committee in accordance with the Australian National Statement on Ethical Conduct in Human Research (2007).

### Materials

#### Virtual Reality Apparatus

The Oculus Go HMD was used [73] to deliver the VR experience to participants. This is a standalone HMD that does not require tethering to a computer or the use of a mobile phone.

#### Virtual Reality Content

YouTube VR (Google LLC) was used for the playback of 360-degree videos. This is an app that is specifically used for viewing YouTube videos in VR HMDs. All 360-degree videos were downloaded to the HMD to reduce reliance on the internet and improve the reliability of playback. Wander (Parkline Interactive) was used to navigate to places of interest tailored to the participant. This app uses data from Google Street View and has the feature of saving places visited for future access.

#### Questionnaire for Establishing Virtual Reality Content

An interview was conducted to develop tailored content for each participant and was based on reminiscence therapy guidelines [24]. Questions included the following: “What significant memories do you have from your childhood?” “What significant life events do you have positive memories of?” and “What memories do you have from working, including details about your first job, and work in the home?”

#### Psychogeriatric Assessment Scale

Cognitive status was obtained from the current records of the residential aged care facility using the PAS [74]. The PAS includes both a subject and informant interview and provides a summary of functioning related to cerebrovascular disease, cognitive status, and behavioral disturbance that can occur in dementia [74]. Scores range from 0 to 21 with a score of ≥4 indicating likely cognitive impairment [75] and the risk of further cognitive decline [76].

#### Apathy Evaluation Scale Self-Rated Version

Apathy was measured using the AES [4]. This was included to measure the participants’ current level of apathy. The AES contains 18 items measured on a scale ranging from *not at all*, *slightly*, *somewhat*, and *a lot*. The AES scores range from 18 to 72, with higher scores indicating a higher level of apathy. Rating is based on the previous 4 weeks. The self-rated version of the AES has been found to have satisfactory test-retest reliability of .76 and good internal consistency (*α*=.86) [4]. The internal consistency for this study was *α*=.77. Questions included, “Are you interested in having new experiences?” and “Do you spend time doing things that interest you?”

#### Simulator Sickness Questionnaire

Side effects were measured using the SSQ [77]. This questionnaire is widely used in VR-related research [78]. It consists of 16 items that assess the symptoms in the VR environment using a 4-point rating scale from 0 (indicating *no symptoms*) to 3 (indicating *severe symptoms*). The SSQ has 3 subscales, including nausea, oculomotor, and disorientation, and has demonstrated good internal consistency (*α*=.87) [79]. Internal consistency for this study was *α*=.69. Both the total and subscale SSQ scores require weighting. The subscale scores were weighted as follows: nausea 9.54, oculomotor 7.58, and disorientation 13.92. Total scores were calculated by adding the 3 unweighted subscale scores and multiplying by 3.74. The 3 subscales include overlapping symptoms, for example, the symptom of *blurred vision* overlaps both the oculomotor and disorientation subscales. Symptoms measured included *headache*, *nausea*, *sweating*, and *eye strain*.

#### Slater-Usoh-Steed Presence Questionnaire

The Slater-Usoh-Steed Presence Questionnaire (SUS) was used to assess how realistic participants thought the VR environment was [80]. The SUS consists of 6 items scored from 1 to 7. Questions include “To what extent were there times during the experience when the virtual environment was reality for you?” and “During the time of your experience, did you often think to yourself that you were actually in the virtual environment?” This scale has demonstrated acceptable internal consistency (*α*=.75) [81]. Internal consistency for this study was *α*=.74.

#### Verbal Fluency

Both phonemic and semantic verbal fluency tasks were administered to the participants [82]. In the phonemic verbal fluency task, participants had to name as many words as possible starting with the letter *F* or *P*. In the semantic verbal fluency task, participants had to list as many words as possible in the category of either *animals* or *fruit/vegetables*. The tasks were timed for 1 min each.

#### Expectations/Enjoyment Measure

Participants were asked about their expectations before the VR experience “What are your expectations about the VR experience, in reference to overall enjoyment?” After the VR experience, they were asked, “How much did you enjoy the experience?” Each question was given a rating ranging from 1 to 10.

#### Debriefing Questionnaire

A debriefing interview consisting of 8 questions at the end of the VR session (session 2) assessed the feedback from the participants. Interview questions included “Did you find the experience enjoyable?” and “What did you like about the experience?”

### Procedure

The study consisted of 2 sessions, with each session lasting for approximately 60 min. Before consent, to ensure that the participants were able to see images satisfactorily in the headset, a short demonstration was performed where participants viewed images in the HMD using the Wander app. Once it was established that the participants could satisfactorily see the images in the headset, and they consented to participate in the study, an appointment was made for the first session. In the first session, basic demographic data were collected. Participants were then asked a series of questions (questionnaire for establishing VR content) to establish topics for sourcing content to be viewed in the HMD. Finally, the participants completed the AES.

Once the researcher had sourced content based on data gathered from the interview in the first session for viewing in the HMD (approximately 3 days apart), an appointment was made for the second session. Before and after the VR session, 2 verbal fluency tests (phonemic and semantic), expectations/enjoyment rating, and the SSQ were administered. Alternate forms of verbal fluency tests were counterbalanced for each participant. Although the SSQ was designed to be used postexposure to VR, it was decided to follow the precondition of screening participants who may have health-related conditions [77]. The sourcing of content was completed between the first and second sessions and took approximately 90 min for each participant. Participants viewed the VR content that was timed for 20 min. The researcher determined the order of the content being viewed, and during the VR session, a conversation occurred between the participant and the researcher about the participant’s past experiences with regard to the content. Participants were not given access to the controllers, and all navigation was performed by the researcher. This was done to minimize frustration and provide a seamless experience for each participant. The HMD image was mirrored to a laptop to enable the researcher to see what the participant was viewing and to allow the researcher to navigate within the apps. Participants remained seated during the VR content exposure, as standing has been found to increase the risk of motion sickness when using HMDs [83]. Remaining seated also reduced the risk of falls while wearing an HMD. A swivel chair was used to enable participants to turn freely when viewing the VR content, thereby limiting neck movement. One participant was restricted to a mobile chair, and the VR content was delivered while they were lying down at a 45-degree angle in the chair. The researcher turned the mobile chair during VR exposure to enable the participant to view the 360-degree videos. Another participant with mobility issues was seated in a lounge-type chair in their room. After VR exposure, the SUS was administered as a measure of presence, followed by the debriefing questionnaire.

## Results

Separate Bonferroni corrections for multiple comparisons were applied for each family of tests. For the 2 fluency tasks, alpha was set at <.025 (.05/2). For the 4 SSQ scores, alpha was set at <.013 (.05/4). For the pre-expectations and enjoyment scale, alpha was set at <.05 (.05/1). Assumptions of normality using the Shapiro-Wilk test found both oculomotor and disorientation subscale SSQ scores to significantly deviate from a normal distribution; therefore, the Wilcoxon signed-rank test was performed for all SSQ comparisons. Two-tailed paired samples *t* tests were used for the remaining comparisons. Means and standard deviations for all pre- and postmeasures are shown in Table 1. Two-tailed paired sample *t* tests revealed that semantic verbal fluency scores were significantly higher post-VR, which was the only significant difference (Table 1).

Neither pre- nor post-VR comparison between phonemic and semantic fluency reached statistical significance in a two-tailed paired samples *t* test (pre-VR: mean 0.40 (SD 5.10); 95% CI −2.42 to 3.22; t_14_=0.30; *P*=.77. post-VR: mean −2.47 (SD 5.21); 95% CI −5.35 to 0.42; t_14_=−1.83; *P*=.09).

Bivariate (Pearson) correlations between the AES scores with either phonemic or semantic verbal fluency taken at baseline were not significant (AES and phonemic: *r*=0.309; 95% CI −0.241 to 0.709; *P*=.26; AES and semantic: *r*=−0.104; 95% CI −0.585 to 0.432; *P*=.71).

**Figure 1:**
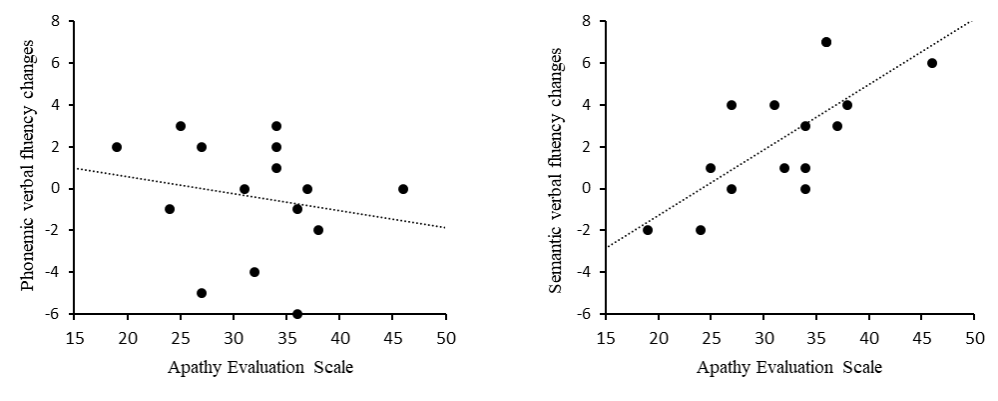
Correlation between scores on the Apathy Evaluation Scale at baseline and postminus pre–virtual reality session semantic and phonemic verbal fluency. Two participants scored the same in the semantic verbal fluency correlation.

Bivariate (Pearson) correlations between the AES scores taken at baseline and phonemic verbal fluency change scores postminus pre-VR were not significant (*r*=−0.195; 95% CI −0.643 to 0.352; *P*=.49. There was a strong positive relationship between the AES and semantic verbal fluency change scores postminus pre-VR (*r*=0.719; 95% CI 0.327 to 0.900; *P*=.003; Figure 1).

**Figure 2:**
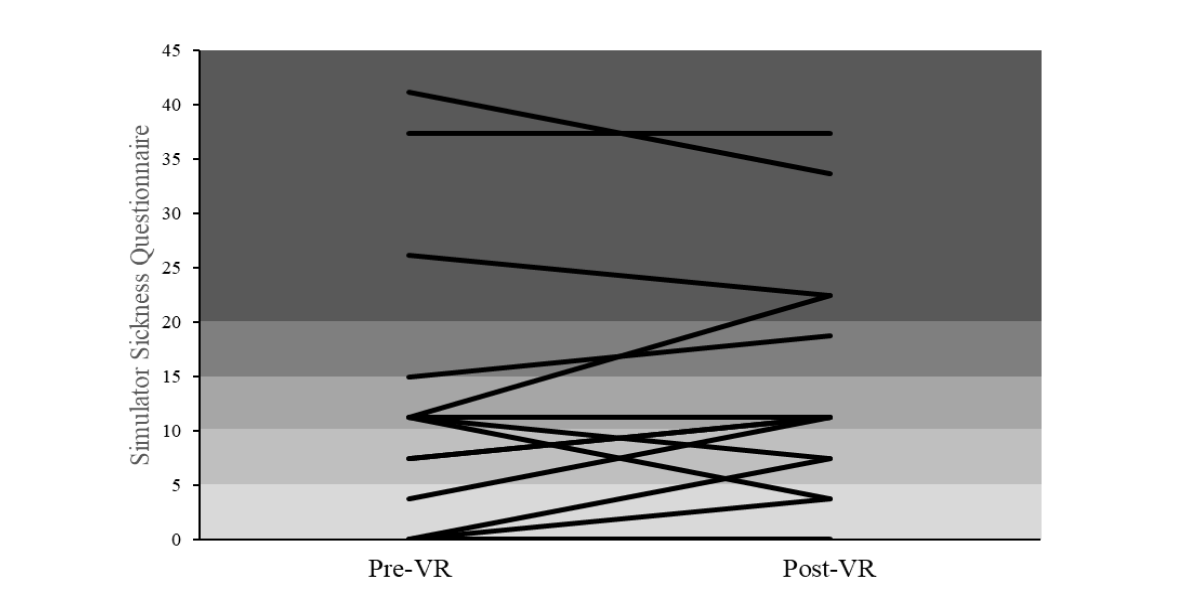
Pre– and post–Simulator Sickness Questionnaire scores with each line representing 1 participant. Shaded areas indicate Simulator Sickness Questionnaire score cutoffs of <5, 5 to 10, 10 to 15, 15 to 20, and >20. VR: virtual reality.

**Figure 3:**
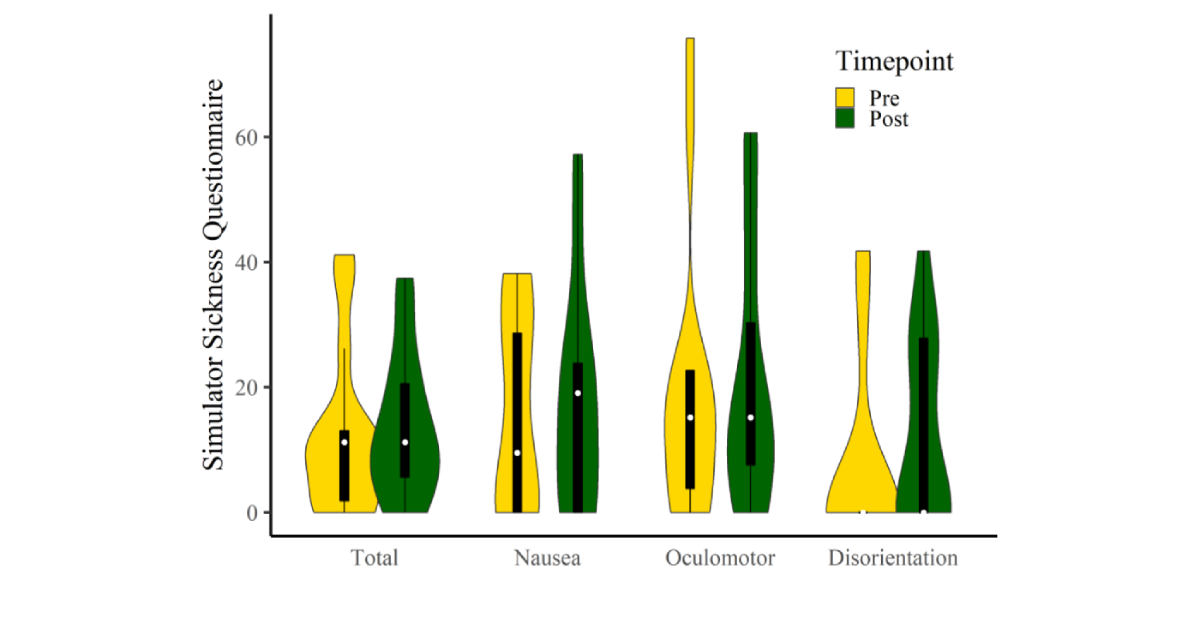
Distribution of Simulator Sickness Questionnaire total and subscale scores at pre- and post–virtual reality intervention, indicated by boxplots within violin plots, white dots represent medians.

**Figure 4:**
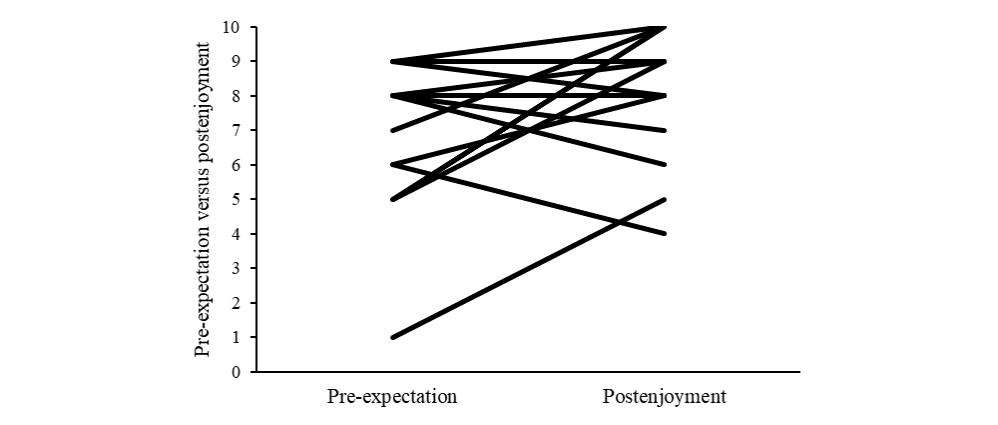
Pre–virtual reality expectations versus post–virtual reality enjoyment scores with each line representing one participant.

Correlations between the AES scores and either phonemic or semantic change scores were significant (*P*=.03). Statistical differences between correlations were calculated using the Steiger method [84]. Figure 2 shows a breakdown of the SSQ scores for each participant pre- and post-VR using SSQ score cutoffs [85]. Figure 3 shows a summary of the total and subscale SSQ scores. Figure 4 shows a breakdown of the pre-expectation/postenjoyment scores for each participant.

**Table 1:** Means and standard deviations for all pre- and post-VR measures with statistics and effect size.

Measures	Pre-VR^a^, mean (SD)	Post-VR, mean (SD)	*t* statistic (*df*)	*P* value	*d* value
Phonemic verbal fluency	10.13 (3.78)	9.73 (3.81)	0.55 (14)	.59	0.142
Semantic verbal fluency	9.73 (4.10)	12.20 (4.54)	−3.27 (14)	*.006* ^d^	−0.843
SSQ total^b^	12.22 (13.07)	13.46 (11.38)	24.00^c^	.45	−0.230
SSQ nausea	15.90 (15.99)	17.17 (17.74)	14.00^c^	.62	−0.107
SSQ oculomotor	18.70 (21.98)	20.72 (20.75)	8.50^c^	.40	−0.174
SSQ disorientation	7.42 (15.67)	12.06 (14.76)	1.00^c^	.10	−0.408
Expectations/enjoyment	6.80 (2.18)	8.07 (1.87)	−2.02 (14)	.06	−0.520

^a^VR: virtual reality.

^b^SSQ: Simulator Sickness Questionnaire.

^c^Denotes the Wilcoxon signed-rank test. The remaining comparisons are paired samples two-tailed *t* tests.

^d^Significant value shown in italics.

Most participants had some difficulty with the SUS questionnaire; question number 5 had to be repeated or further explained. Four participants did not attempt answering question number 5, and 2 participants could not complete any of the questions. Therefore, data from the SUS were not analyzed.

After the VR session, the participants were asked a set of 8 questions to gain feedback about their experiences. Although some of the questions could be answered with a yes or no response, participants were given the opportunity to elaborate on their answers, if required.

All the study participants found the experience enjoyable. When asked what they liked about the experience, 11 participants said they enjoyed reminiscing, 4 participants reported it was nice to do something different, 1 participant reported how realistic the experience was, and finally, 1 participant liked the colors and clarity of the images shown. Comments included “liked the accuracy, feeling of being there and sites that I wanted to see again,” “taken back to somewhere that I know I will never see again,” “better than looking at a computer,” and “I had a feeling of being there.”

In reference to acceptability, 1 participant reported that the HMD was heavy; however, this was rectified by adjusting the head strap. All remaining participants found everything acceptable. When asked if they would do it again, all responded yes, with 1 participant stating, “only for research purposes.”

There were instances where participants reported negative symptoms or side effects. This included 1 participant with discomfort around the cheekbone and forehead areas. Another participant reported dizziness and the feeling that they were “going to fall off the edge;” a further 2 participants reported the feeling that they were going to fall off the edge. One participant reported eyestrain, and another participant reported nausea 10 min after the VR session, which subsided within 10 min. Therefore, 6 out of 17 participants reported some type of negative symptoms or side effects.

Participants were asked to offer suggestions to improve their experience. One participant stated that they would have liked to use the controller to navigate in the virtual environment on their own; another participant suggested having some type of interaction; a third participant said it would have been good to “show the difference between then and now,” referring to the Wander app; and finally, one participant would have preferred to see the places that they had not been to before. All participants said they would recommend the VR experience to a friend.

Finally, the participants were asked what they thought about the questionnaires. A total of 12 participants reported that they were all acceptable; 1 participant did not like them without giving a reason; 1 participant could not remember enough to comment; 1 participant said there were too many questions; 1 participant thought there was not a lot of relevance; and 1 participant said that the pre-VR measures did “cause some tension.”

## Discussion

This study found that VR using HMDs to deliver reminiscence therapy is feasible and may be beneficial for aged care residents with apathy. Of the 20 participants who were requested to participate, 17 agreed, indicating a willingness of residents in aged care to participate in research using VR technology. Therefore, the number of available participants for this study was easily achieved. The 3 participants who declined to participate were not interested. It is not known how often exclusion criteria had to be applied, as participants were selected by senior staff at the residential aged care facility following the exclusion criteria. A challenge for future research in apathy is a possible reluctance of those with high levels of apathy to participate because of a lack of interest and motivation, similar to reduced involvement in therapeutic activities found in those with higher levels of apathy [86]. The response rates to measures used in this study were positive. The SUS was the only measure that caused difficulties in understanding the questions, with 2 participants not completing any of the questions. The SUS was chosen because of the small number of items (6) and quick completion time compared with other measures of presence [81] to reduce participant burden. It was validated in a younger sample of university-based students with a technical background, who had no difficulties in completing the questionnaire [81]. As per the qualitative feedback received by this study, older adults may have difficulties in understanding some questions of the SUS. All other measures were fully completed, and all participants adhered to the procedure of the research. It should be noted that the reliability values for measures reported in the literature may not generalize to this study because of sample differences, that is, older adults with neurological and other chronic diseases.

The time taken to create content varied depending on the participant’s previous experiences. In this study, the researcher prepared the content before the VR session. It was found that by using readily available apps, the content could easily be sourced for all participants for a VR exposure time of 20 min. This finding is of importance for this study, as individualized reminiscence therapy can be resource intensive [87]. The accessibility of content utilizing the apps in this study reduces the resources required for creating content and places fewer demands on staff if applied in aged care lifestyle activities.

In this study, the preparation of VR content took approximately 90 min for each participant. This time can be substantially reduced once a library of 360-degree travel or music videos has been created. In addition, in this study, the content was compiled before the VR session to maximize the amount of time the participant would reminisce. Sourcing of VR content could also be done during a reminiscence session, working together with the participant while they are viewing images in the HMD, and an area for future research. Working together with the participant not only reduces the preparation time but also allows sessions to be tailored and flexible depending on participants’ responses [88]. Traditional methods, including making up a life history book, can require substantial resources and are also limited [88]. Using the Wander and YouTube apps reduces the preparation time and provides a wide range of content that can be easily changed. The use of a group reminiscence therapy format that is more generic can be a preferred approach because of the ability to improve social interaction [89] and can also be less resource intensive [90]. However, each person has a unique history, and using individualized content has been demonstrated to be more effective than using generic content [91]. In addition, an individualized approach is person-centered, focusing on a person’s strengths, thereby helping maintain a sense of self and identity [92].

No technical problems with conducting the sessions were encountered. To enable successful delivery of the content, it is recommended that the VR headset be mirrored to an external screen and controlled by the person delivering the content. Thus, the focus is on viewing the content rather than trying to learn to navigate or use the controllers. Although 2 participants would have liked to interact in some form with the virtual environment, this study was designed to provide a seamless experience. As the VR exposure consisted of one session, the intention was to reduce any frustration that could occur because of pressing the wrong buttons on the controller. We cannot draw any conclusions about the extent to which aged care residents could navigate within the app and use the controllers themselves, as this was not attempted. However, the feasibility of controller usage and navigation in VR in this population is an avenue for future research for studies conducted over a longer period. Providing the ability to interact allows the user to act independently and may improve the sense of presence. Recent research has found that the use of VR in providing interaction may be an alternative way of delivering stimulation to people with dementia who do not participate in other lifestyle activities [93].

Verbal fluency was used as a proxy measure to assess improvements in apathy. Both semantic and phonemic verbal fluency results were similar at baseline, despite phonemic fluency being known to be more difficult [94]. This could be because of the level of education in this group of participants (mean 13 years, SD 3), as higher education has been correlated with increased performance in phonemic fluency [95]. In pre-VR sessions, both phonemic and semantic fluency scores were not associated with the participants’ level of apathy. In post-VR sessions, not only did semantic fluency increase but the results were positively correlated with participants’ level of apathy at baseline. This suggests that the greatest cognitive improvements were seen in participants with higher levels of apathy. What is unclear is why these differences were seen only in semantic fluency. Similar improvements in verbal fluency have been reported in a study using traditional reminiscence therapy in a sample of dementia patients [96] in a short number of sessions. However, in this study, increases were seen in both phonemic and semantic fluency, and apathy was not measured. The executive function of initiation required for verbal fluency may be low in persons with apathy [97], and the reliving of autobiographical memories in an immersive environment may have stimulated the process of initiation.

The results from the debriefing questionnaire were positive and in agreement with previous research, finding acceptance of using HMDs in older adults [46-48]. This study further expands the knowledge of acceptability by demonstrating that VR using HMDs can be used to deliver reminiscence therapy with participants of varying levels of cognitive impairment, including minimal, mild, and moderate impairment, as assessed by the PAS. All participants reported enjoying the VR experience, despite 6 participants reporting a side effect. Another indicator of positive response was that all participants stated they would do it again (although 1 said “for research purposes only”). The most common response about what the participants liked about the experience was related to reminiscence and the enjoyment of seeing places from their past, indicating that this is something this age group wants to experience in a VR environment. An important aspect of reminiscence is maintaining a person’s sense of identity [87]. As loss of identity can be a key feature of Alzheimer disease that can increase apathy [98], participating in tailored reminiscence therapy may help in maintaining a person’s sense of self and identity and therefore improve levels of apathy. The expectations questions asked pre-VR compared with the enjoyment level post-VR, although in a positive direction, failed to reach a significant level. There were 2 participants in pain because of pre-existing conditions during the VR session, which may have contributed to this result.

Although quantitative findings of short-term side effects from VR were not significant, the debriefing questionnaire raised some concerns. Six participants reported side effects, 4 of which were symptoms related to items measured by the SSQ, including nausea, eyestrain, and dizziness. These symptoms dissipated quickly. In one instance where a participant experienced nausea, it occurred 10 min after the VR experience with symptoms subsiding within 10 min. This after-effect highlights the importance of monitoring participants both during and after VR to avoid health and safety implications.

Moreover, 3 participants had the feeling of “falling off the edge” in VR. This symptom was not captured by the SSQ and did create some discomfort, with 2 of the participants requesting to be moved back to their original position, when they had not actually moved. This could not be associated with the type of content. One explanation could be that sometimes the camera shooting the VR content is elevated, and a high sense of presence may have given a realistic feeling of their position in the virtual environment. Another factor that may have contributed to this symptom is the common occurrence of balance problems in older adults with neurological diseases [99]. This symptom will need to be monitored in future studies when working with this population.

The importance of taking pre- and post-SSQ measures was demonstrated in this study, with some participants reporting symptoms before the commencement of VR. This was usually because of pain from existing conditions. It is also interesting to note that in 4 participants, the SSQ scores decreased post-VR; this included a participant with the highest pre-VR SSQ score. This reduction in the SSQ scores could be a similar effect seen in the successful use of VR for distraction-based therapies [44,45]. Despite all participants finding the experience enjoyable, it is important to consider that 6 out of 17 participants (35%) had some type of negative response when viewing the VR content and the importance of monitoring symptoms. When using cutoff scores for the SSQ (negligible symptoms: <5, minimal symptoms: 5-10, significant symptoms: 10-15, concerning symptoms: 15-20, and problematic symptoms: >20 [85]), it can be seen in Figure 2 that the majority of participants scored >5, with 4 participants experiencing problematic symptoms (>20). However, these cutoffs were created with military personnel using flight simulators, and how these cutoff scores relate to the general population and, in the case of this study, older adults is unknown.

This study aimed to assess feasibility; therefore, a control group was not included, and we could not compare results using a traditional reminiscence therapy approach. However, the aim was first to establish if HMDs could be used in this population for reminiscence therapy. Although alternate forms of both phonemic and semantic verbal fluency were used to reduce practice effects, the lack of a control group receiving no VR intervention warrants a caveat when interpreting the results. It must remain open to what degree the pre-post comparisons are the result of the VR exposure versus a pure retest effect. It is also not known how long the verbal fluency improvements would last and if other aspects of apathy were reduced. Anecdotally, staff members providing services to participants involved in the research reported positive mood changes during the research process, and there were also instances where positive changes in behavior were reported by family members of participants involved in the research.

As the self-rated version of the AES was used, it needs to be considered that people with apathy will normally underestimate their condition [4]. Therefore, true scores may be higher than reported. This study did not specifically recruit participants with apathy, and a challenge with future research is the difficulty of recruiting participants who do have apathy because of their lack of interest and motivation.

This study demonstrated that VR using HMDs can be used to deliver tailored content about a person’s past and that residents in aged care enjoyed this experience. The aged care site in this study was in an area of high socioeconomic status, and study participants had a relatively high education level. Higher levels of education have been associated with an increased willingness to participate in research [100]. The sample was also selected by the staff and therefore was not a random sample, possibly introducing a selection bias [101]. How these factors would generalize to a random sample with an average or lower education level is unknown and needs to be considered when determining generalizability as participation rates, adherence to sessions, and therefore feasibility may differ. Immediate effects on cognition for those with higher levels of apathy after 1 session is an interesting finding and highlights the importance of continuing this research. The VR experience can be implemented and incorporated into current lifestyle activities in aged care with the correct protocols in place. VR is being increasingly used in older adults in areas including assessment, cognitive training, and rehabilitation; therefore, it is critical to monitor side effects, particularly after the VR experience, to avoid any health and safety implications and assess whether the benefits of using HMDs outweigh the risk of side effects. This study provides initial evidence that it is feasible to use VR with HMDs for therapy to treat symptoms of apathy in older adults in residential aged care. Further research over multiple sessions using an active control group to compare with traditional reminiscence therapy will help establish the advantages of using VR with HMDs.

## Abbreviations

AES: Apathy Evaluation Scale
HMD: head-mounted display
PAS: Psychogeriatric Assessment Scale
PTSD: post-traumatic stress disorder
SSQ: Simulator Sickness Questionnaire
SUS: Slater-Usoh-Steed Presence Questionnaire
VR: virtual reality
